# Effects of Autologous Blood‐Derived Extracellular Vesicles on Skin Regeneration and Anti‐Aging: A Clinical Study

**DOI:** 10.1111/jocd.70760

**Published:** 2026-02-22

**Authors:** Juyan Song, Chenyu Zhao

**Affiliations:** ^1^ Department of Dermatology Guiyang First People's Hospital Guiyang China; ^2^ School of Pharmacy Queen's University Belfast Belfast UK; ^3^ Department of China Medical University‐The Queen's University of Belfast Joint College, School of Pharmacy China Medical University Shenyang China

**Keywords:** autologous blood‐derived extracellular vesicles, neocollagenesis, skin rejuvenation

To the Editor,

We read with great interest the recent original article by Kang et al. regarding the clinical efficacy of autologous blood‐derived extracellular vesicles (EVs) for skin rejuvenation [1]. As the paradigm of aesthetic medicine shifts from volume replacement to biological regeneration, studies investigating autologous therapies are pivotal [2]. While the authors report statistically significant improvements in wrinkle depth and skin lifting, we believe several methodological aspects warrant rigorous discussion to contextualize these findings within established physiological and research standards.

A primary point of consideration is the reported timeline of clinical improvement. The study notes significant reductions in wrinkle depth and enhancements in skin lifting as early as 5 days post‐treatment [1]. Current understanding of cutaneous wound healing physiology suggests that the proliferative phase, characterized by fibroblast activation and neocollagenesis, typically spans several weeks [3]. The synthesis and maturation of types I and III collagen into a structured matrix generally require months to manifest visible structural changes. Consequently, the immediate volumetric restoration and lifting effects observed at Day 5 may be attributed, at least in part, to post‐injection edema or the hydrostatic volume of the carrier fluid rather than de novo tissue regeneration. Differentiating between transient volumetric changes caused by tissue trauma and true biological remodeling is critical. Extended follow‐up periods beyond 3 weeks would clarify the durability of these effects once the initial inflammatory response subsides.

Furthermore, the study design utilized a single‐arm approach where participants served as their own controls [1]. In the absence of a vehicle control group receiving saline or EV‐depleted plasma, it becomes challenging to isolate the specific therapeutic contribution of the extracellular vesicles from the non‐specific effects of microneedle injury (mesotherapy effect) or plasma proteins remaining in the isolate. The placebo effect and observer bias in open‐label aesthetic studies are also well‐documented factors that can influence subjective satisfaction scores.

Regarding the isolation methodology, the authors employed size‐exclusion chromatography (SEC). While SEC is a robust technique, recent guidelines from the International Society for Extracellular Vesicles (MISEV2023) highlight the difficulty of separating EVs from lipoproteins, particularly chylomicrons and very‐low‐density lipoproteins (VLDL), which overlap in size with small EVs [4]. As blood plasma is abundant in lipoproteins that can also carry bioactive molecules and microRNAs, characterizing the isolate for specific lipoprotein markers (such as ApoB) would strengthen the claim that the observed clinical benefits are exclusively mediated by EVs. Without reporting the ratio of EV markers to contaminants, the purity and specific potency of the preparation remain uncertain.

We commend Kang et al. for contributing valuable data to this rapidly evolving field. Future investigations incorporating randomized, split‐face designs with longer follow‐up intervals and rigorous adherence to MISEV characterization standards will be essential to definitively establish the efficacy of autologous EV therapies.

## Author Contributions

All authors extensively discussed the original manuscript. J.S. and C.Z. conceptualized and wrote the letter. All authors reviewed and approved the final version of the manuscript.

## Funding

The authors have nothing to report.

## Ethics Statement

The authors have nothing to report.

## Conflicts of Interest

The authors declare no conflicts of interest.

## Data Availability

Data sharing not applicable to this article as no datasets were generated or analysed during the current study.
